# Acupuncture for chronic neck pain: a pilot for a randomised controlled trial

**DOI:** 10.1186/1471-2474-7-99

**Published:** 2006-12-09

**Authors:** Gemma C Salter, Mark Roman, Martin J Bland, Hugh MacPherson

**Affiliations:** 1 Department of Health Sciences, University of York, York, UK; 2 York Medical Group, Selby & York Primary Care Trust, York, UK; 3 Foundation for Traditional Chinese Medicine, York, UK

## Abstract

**Background:**

Acupuncture is increasingly being used for many conditions including chronic neck pain. However the evidence remains inconclusive, indicating the need for further well-designed research. The aim of this study was to conduct a pilot randomised controlled parallel arm trial, to establish key features required for the design and implementation of a large-scale trial on acupuncture for chronic neck pain.

**Methods:**

Patients whose GPs had diagnosed neck pain were recruited from one general practice, and randomised to receive usual GP care only, or acupuncture (up to 10 treatments over 3 months) as an adjunctive treatment to usual GP care. The primary outcome measure was the Northwick Park Neck Pain Questionnaire (NPQ) at 3 months. The primary analysis was to determine the sample size for the full scale study.

**Results:**

Of the 227 patients with neck pain identified from the GP database, 28 (12.3%) consenting patients were eligible to participate in the pilot and 24 (10.5%) were recruited to the trial. Ten patients were randomised to acupuncture, receiving an average of eight treatments from one of four acupuncturists, and 14 were randomised to usual GP care alone. The sample size for the full scale trial was calculated from a clinically meaningful difference of 5% on the NPQ and, from this pilot, an adjusted standard deviation of 15.3%. Assuming 90% power at the 5% significance level, a sample size of 229 would be required in each arm in a large-scale trial when allowing for a loss to follow-up rate of 14%. In order to achieve this sample, one would need to identify patients from databases of GP practices with a total population of 230,000 patients, or approximately 15 GP practices roughly equal in size to the one involved in this study (i.e. 15,694 patients).

**Conclusion:**

This pilot study has allowed a number of recommendations to be made to facilitate the design of a large-scale trial, which in turn will help to clarify the existing evidence base on acupuncture for neck pain.

## Background

It is estimated that during the course of a year, approximately 34% of adults experience neck pain, of which a significant proportion is chronic, with about 14% experiencing neck pain for at least six months duration [1]. As well as the impact it has on individuals at a personal level, there are also significant financial costs associated with neck pain [2]. In general, evidence is lacking to support many standard treatments for chronic neck pain [3], and increasingly people are using alternative treatments such as acupuncture [4,5].

A systematic review on the effectiveness of acupuncture treatment for neck pain found the evidence to be inconclusive [6], with some studies producing positive results in favour of acupuncture and others producing negative or equivocal findings. In their systematic review, White and Ernst [6] documented the poor quality of the reviewed trials, none of which scored full points on the quality assessment used in the review. These findings are reinforced by White et al [7], who discouraged further reviews on the subject, and argued instead that "the focus must be on actually conducting more clinical trials" of good quality, a view echoed by others in the field [6,8-10].

Since White and Ernst's review [6], a number of trials have been conducted. Although they vary considerably in terms of their design, the findings remain inconsistent, with most studies showing some positive results in favour of acupuncture [4,11-15], some showing negative results [16,17], and others showing neutral/equivocal findings [18,19]. An examination of these more recent studies reveals the continued existence of many of the methodological difficulties referred to in previous reviews [6,7].

In this pilot study, we have focused on establishing the key design features required for a full scale randomised controlled trial of the effectiveness of acupuncture for chronic neck pain, where acupuncture is provided as an adjunct to usual GP care. In particular we aimed to establish: the potential recruitment rate; the level of attendance for, and acceptability of, acupuncture to patients; the variability in the primary clinical outcome measure; and the loss to follow-up. A key outcome of this pilot is the sample size calculation for the full-scale trial. As part of this pilot, we interviewed patients, acupuncturists and GPs to facilitate a fuller understanding of patient and practitioner perspectives, however this qualitative data is not reported here.

## Methods

### Participants

Participants were recruited to the study from one general practice in York, North Yorkshire, using a retrospective method of recruitment, similar to that used by McCarney et al [20]. The general practice conducted a search of its database to identify patients over 18 years of age who had consulted the practice with neck pain in the previous 12 months. Patients were excluded if they were known to have cancer, rheumatoid arthritis or ankylosing spondylitis. All of the identified patients were sent an information pack about the study. Consenting patients were included in the study if they had experienced neck pain in the previous four weeks. Patients were excluded if they: a) had their main pain below the elbow or in some other part of the body (other than the neck); b) had received surgery on the neck; c) had haemophilia; d) were currently receiving acupuncture; e) were awaiting legal action related to their neck pain; or f) were unable or unwilling to provide informed consent. York Local Research Ethics Committee approved the study.

### Randomisation

Participants were registered to the study by the authors, but random allocation was performed by the York Trials Unit, using computer generated random numbers. This allocation was conducted independently of the study researchers and the clinicians involved in the treatment of the patients, although it was not possible to remain blind to allocation once randomisation had been completed. Due to resource constraints and the initial costs associated with acupuncture, unequal randomisation was used, so 10 patients were randomised to receive acupuncture plus usual GP care, and 14 to usual GP care only. For randomisation, participants were stratified according to whether they had a higher or lower neck pain score on the NPQ.

### Interventions

The acupuncture intervention consisted of up to 10 sessions, with a treatment protocol based on a standardised framework, with both fixed and variable components (see Additional file 1 for further details). The variable components were driven by the theoretical underpinnings of traditional Chinese medicine, including the Five Element approach. The protocol incorporated active management techniques (such as devising a treatment plan with patients, providing relevant explanations, etc.), theoretical frameworks that underpinned diagnosis and treatment, auxiliary techniques (such as acupuncture-related massage), and other aspects of patient care (such as offering lifestyle and dietary advice). Usual GP care was available to both groups, and at three months patients were asked to record all treatments they had received.

### Outcomes

The primary clinical outcome was the Northwick Park Neck Pain Questionnaire (NPQ) measured at three months [21], which is scored out of 36, or 32 for non car drivers, and is presented as a percentage. Medication use at baseline and three months was also collected. Further data, not reported here, was collected regarding quality of life (SF-36) and health utility (EQ-5D). At three months, satisfaction with treatment was measured in both groups, using questions relating to satisfaction with information provided, the treatment received, and the overall care received. Adverse events were also monitored in the acupuncture group.

### Data analysis

The outcome data was analysed using SPSS Windows, version 12. Analysis of the primary outcome data was on an intention to treat basis and carried out using ANCOVA with the treatment group as the independent variable, and the baseline score as a covariate. The sample size calculation was based on the minimum clinically meaningful change to the NPQ. The residual variance was estimated from regression on baseline and treatment allocation, a method equivalent to that proposed by Frison & Pocock [22]. This variance was inflated by the method of Browne [23], using an upper one-sided 90% confidence interval.

## Results

### Recruitment rate and baseline characteristics

**Figure 1:**
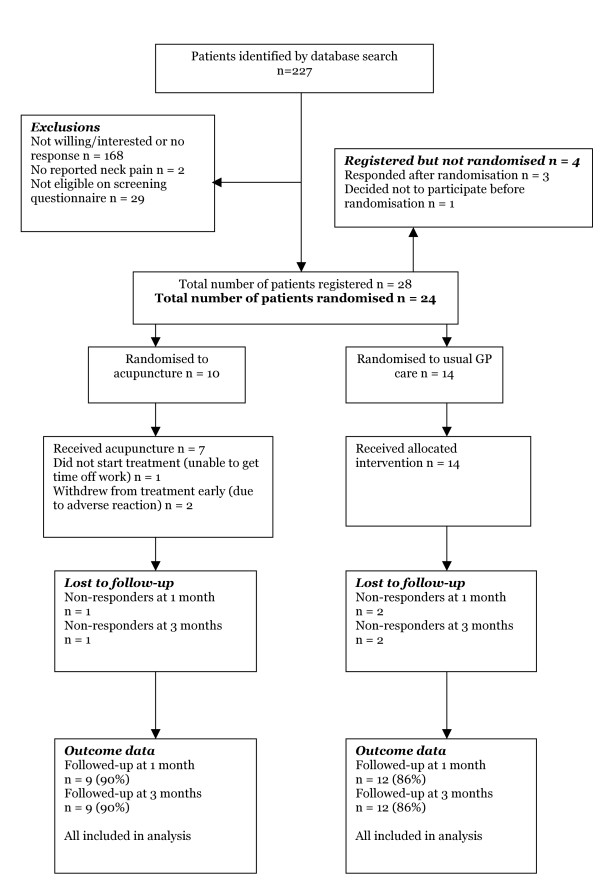
Patients' progress through the trial.

Of the 15,694 patients on the GP database, 227 were identified in April 2005 as having consulted with neck pain in the previous year, i.e. 1.4% of the practice population (see Figure 1). Read Codes included cervicalgia (65%), cervical spondylosis (10%), whiplash (8%), wry neck torticollis (5%), neck sprain (4%), and stiff neck (3%). Of those identified, 28 patients (12.3% of identified patients) were eligible to participate in the trial. However, three patients responded after the cut-off date (3 weeks after identification), and one patient withdrew consent prior to randomisation. Therefore only twenty-four patients (10.6%) were randomised, ten to acupuncture and fourteen to usual care. At baseline, both groups had no significant differences in characteristics which are reported in Table 1.

### Acupuncture treatment

**Figure 2:**
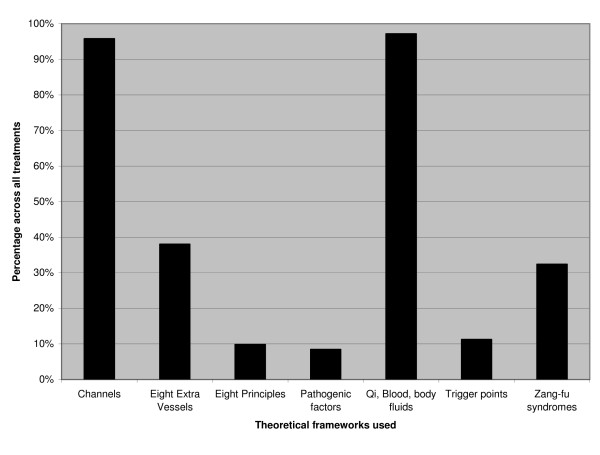
The frequency of the theoretical frameworks used by practitioners across all acupuncture treatments.

Within the trial, acupuncturists completed treatment logs, providing detailed descriptions of the acupuncture treatment and the theoretical frameworks used (see Figure 2). Acupuncturists used between 5 and 24 needles per treatment, and the needles were between 13–50 mm in length, with a gauge of between 0.18 to 0.36 mm, and a depth of insertion between 0.2 to 2.5 cm. The most commonly used points were GB-21 (used in over 90% of treatments), followed by Ah Shi points (78%), GB-20 (57%), Huatuojiaji at C6 (the sixth cervical vertebra) (47%), S-I3 (47%), and Huatuojiaji at C7 (42%). Acupuncturists also used auxiliary techniques, primarily acupressure massage (72%) and offered lifestyle support regarding relaxation (16%), diet (13%), exercise (11%) and rest (9%).

### Attendance and acceptability of acupuncture to patients

In the acupuncture group, patients received an average of 7.9 treatments from four acupuncturists. Because of an inability to take time off work, one patient did not receive any acupuncture treatment. Within the trial no serious adverse effects of acupuncture were reported, although patients experienced mild reactions (temporary worsening of symptoms (n = 6), dizziness (n = 6), and tiredness (n = 4)). Two patients withdrew from treatment early due to adverse reactions, both reporting a temporary worsening of symptoms and dizziness, and one also experiencing tiredness as a result of treatment. Positive reactions to acupuncture were also reported including feeling relaxed (n = 6), and feeling energised (n = 2). When comparing the two groups, a higher proportion of patients in the acupuncture group were "very satisfied" or "somewhat satisfied" with treatment (see Table 2), although there were no statistically significant differences between the groups. With the exception of one patient, those receiving acupuncture treatment reported high levels of acceptability.

### Usual GP care in both groups

**Figure 3:**
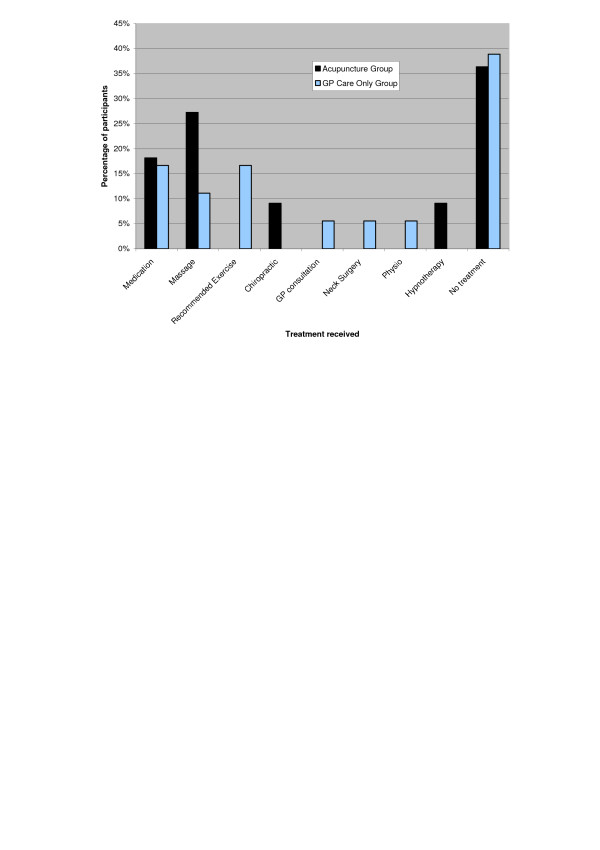
Usual care treatments received at three months as reported by both groups of patients.

At three months, the most commonly received treatments in the usual care group were medication, massage and recommended exercise (see Figure 3). Medication and massage were also common in the acupuncture group. The proportion of patients receiving no treatment (the largest category) was similar in both groups. None of the patients reported receiving any additional acupuncture (other than that provided as part of the study).

#### Variability in the primary clinical outcome measure and loss to follow up

The variability in patients at baseline and at three months, as shown by the standard deviation, is presented in Table 3. At three months, both groups showed an improvement in unadjusted scores on the NPQ, but as expected from such a small sample, there was no significant difference between the groups. When piloting the analysis for the full-scale trial, and controlling for baseline score on the NPQ, the adjusted difference between the means of the two groups was -1.75 percentage points in favour of the acupuncture group, although this difference was not statistically significant (t = -.311, p = 0.759). At three months, a similar proportion of patients in the GP care only group continued to use medication when compared to baseline, but there was a marked reduction in medication use in the acupuncture group. However, a logistic regression (with medication use at three months as the dependent variable, and treatment group and baseline medication use as covariates) showed that the difference was not significant. The loss to follow-up rate at three months was similar in both groups: 10% in the acupuncture group and 14% in the usual GP care group.

#### Sample size calculation and recruitment strategy for a full-scale trial

A clinically meaningful change on the Northwick Park Questionnaire is 5 percentage points [24]. In a regression model that included all participants and adjustment for baseline NPQ scores, the residual standard deviation of the NPQ was estimated at 12.7 with 18 degrees of freedom. Taking into account potential sampling bias [23] using an upper one-sided 90% confidence interval for the variance, we estimated a corrected standard deviation of 15.3. Using this estimate in the sample size calculation gives 197 in each arm of the trial, assuming 90% power at the 5% significance level. Allowing for a loss to follow-up of 14%, the actual sample required would be 229 per arm, i.e. a total of 458 patients.

To calculate the total list size of GP practices needed for patient recruitment, we base our estimate on a response rate 12.3% of 1.4% of eligible patients, i.e. 0.172% of the total list. Given our GP practice size of 15,694, we allowed for sampling variation by calculating the upper 80% one sided confidence interval of this response rate to be 0.20%. Hence to recruit 458 patients, we estimate that the total list size would need to be 230,000, roughly 15 practices of an equivalent size to the one used in this pilot.

## Discussion

The pilot study reported here has provided much useful data for the design of a full-scale trial of acupuncture for chronic neck pain. A key finding is that 12.3% of eligible patients on the GP database consented to participate in the trial, a remarkably similar percentage to the 12% found by McCarney et al [20] in a trial of acupuncture for migraine. Based on the experience of our study, this method will enable a large sample to be recruited to a trial relatively quickly, in comparison with prospective recruitment of incident cases. We also found that a sample size of 458 patients and a GP practice population base of 230,000 would be required in a full-scale trial if GP databases are to be used as a source of identification and recruitment of patients. The evidence from our pilot study suggests that this recruitment strategy is feasible.

We found a trend towards higher levels of satisfaction among those patients referred to acupuncture, compared to those receiving usual GP care alone. All of the patients receiving acupuncture treatment reported high levels of acceptability (with one exception). However, there were some concerns about the safety of acupuncture, specifically the negative reactions to treatment. These included a temporary worsening of symptoms, dizziness, and tiredness, with two patients withdrawing from treatment as a result. Although there were no serious adverse events, defined as "events requiring hospital admission, leading to permanent disability, or resulting in death" [25], safety is clearly an important issue that should be carefully considered when developing the design of a full-scale trial. We recommend the provision of adequate monitoring of adverse events and clinical supervision for acupuncturists.

The pragmatic design of the trial was also considered appropriate given the widespread use of acupuncture and the fact that this design facilitates economic evaluations [26]. Trials embedded in real world practice tend to have strong external validity, though often at the expense of weaker internal validity than explanatory trials. Due to the difficulties of blinding in acupuncture research generally, Birch [10] has highlighted the added importance of ensuring that all stages of analysis are blinded, from data-entry to evaluation of the results. Although impractical for the purposes of this pilot, blinding should be implemented in a large-scale trial, to help avoid the potential for bias [10].

The recruitment processes used in this pilot were successful as patients were identified and recruited relatively simply and quickly. One limitation of our screening procedures was that one patient with cancer inadvertently entered the trial. This indicated a potential flaw with the search strategy, and a future large-scale trial should consider including a cancer question as part of the screening questionnaire given to patients, if the intention remains to exclude patients with cancer.

This study did not set out to establish whether there were components of acupuncture that had specific efficacy. Instead our design was a pragmatic one, where we are working towards an evaluation of the impact of the overall package of acupuncture care. A different research question could have been used to determine the relative contributions of components of the treatment. To do this, a 'placebo' or sham acupuncture control would be used, to control for the components of treatment that are not specific to acupuncture, such as time and attention. Sham approaches are not suitable within pragmatic trials, since they are artificial controls that do not model usual practice [27], making it difficult to meaningfully interpret their results [6]. Sham acupuncture approaches are generally problematic since there is evidence that they can produce a physiological effect that may be therapeutic [28,29].

Although the main outcome measure used in this pilot is a validated scale, such self-report measures are subjective. Therefore for a large-scale trial it might be useful to consider including an objective outcome as a secondary measure [30]. Also, given that there is evidence to suggest that preference and belief might influence outcome [31,32], it is recommended that a large-scale trial establishes preference and belief prior to randomisation, so that their potential influence on outcome can be explored in the analysis [31,33].

Overall, in terms of generalisability of the trial, the broad inclusion criteria for recruiting patients made it more likely that the patients in the trial were fairly representative of those typically presenting with chronic neck pain. The acupuncture treatment protocol was also fairly broad, though sufficiently standardised to assist replication. Acupuncturists found it sufficiently flexible to allow them to use an individualised approach, reflecting traditional acupuncture as it is usually practiced. The generalisability of acupuncture treatment was further improved by using more than one acupuncturist in the trial.

## Conclusion

The results of this pilot have provided useful data on key features of a full-scale trial of acupuncture for chronic neck pain. A sample size has been calculated and a feasible recruitment strategy outlined.

## Competing interests

The author(s) declare that they have no competing interests.

## Authors' contributions

GCS was trial co-ordinator overseeing all aspects of the conduct of the study, including design, data collection, analysis and preparation of the manuscript. HM helped design the study, advised on implementation, and helped draft the manuscript with GS. MR advised on the design, conducted the screening of the GP database, and contributed to revisions of the manuscript. JMB assisted with the data analysis and calculation of proposed sample size. All authors read and approved the final manuscript.

## Pre-publication history

The pre-publication history for this paper can be accessed here:



## Supplementary Material

Additional file 1 Treatment protocol. The treatment protocol provides a description of the processes and procedures involved in the acupuncture treatment. Click here for file

## Figures and Tables

**Table 1:** Baseline characteristics of patients by allocation group

	**GP care only**	**Acupuncture**
N	14	10
Female: n (%)	11 (79%)	7 (70%)
Age: years (SD)	45.5 (16.4)	50.80 (17.1)
Neck pain duration in years (SD)	5.5 (5.5)	5.7 (6.4)
% with other health problems	50%	50%
NPQ score as a % (SD)	38.4% (18.6)	34.31% (11.7)
Number (%) using medication	6 (42.9%)	4 (40%)

Note: Higher scores on NPQ indicate greater severity.

**Table 2:** Patient satisfaction with treatment at 3 months

**Satisfaction with:**	**Response**	**Acupuncture (n = 10) %**	**GP care only (n = 14) %**
**Information received**	Very satisfied	70%	0%
	Somewhat satisfied	0%	21%
	Neither satisfied or dissatisfied	10%	14%
	Somewhat dissatisfied	0%	14%
	Very dissatisfied	0%	0%
	Not applicable	10%	29%
	Missing data	10%	21%

**Treatment received**	Very satisfied	60%	0%
	Somewhat satisfied	10%	21%
	Neither satisfied or dissatisfied	0%	21%
	Somewhat dissatisfied	10%	14%
	Very dissatisfied	0%	0%
	Not applicable	10%	21%
	Missing data	10%	21%

**Overall care received**	Very satisfied	50%	0%
	Somewhat satisfied	20%	14%
	Neither satisfied or dissatisfied	0%	36%
	Somewhat dissatisfied	10%	14%
	Very dissatisfied	0%	0%
	Not applicable	10%	14%
	Missing data	10%	21%

Note: Percentages are rounded to whole numbers.

**Table 3:** Unadjusted scores for the Northwick Park Questionnaire (NPQ) and percentage of patients using medication at baseline and at three months

	**GP care only group (n = 14)**		**Acupuncture group (n = 10)**	
**NPQ**				
**At baseline: % (SD)**	38.44 (18.55)		34.31 (11.71)	
**3 months: % (SD)**	25.72 (16.29)		22.73 (18.64)	
				
**Use of medication:**	**Yes**	**No**	**Yes**	**No**
**At baseline: %**	42.9%	57.1%	40%	60%
**3 months: %**	41.7%	58.3%	11.1%	88.9%

Note: Higher scores on NPQ indicate greater severity
