# Supplementary material for: Acupuncture for chronic neck pain: a pilot for a randomised controlled trial
Source: BMC Musculoskelet Disord. 2006 Dec 9;7:99. doi: 10.1186/1471-2474-7-99 (PMC1713236; doi:10.1186/1471-2474-7-99)
Supplement: Additional file 1 — Treatment protocol. The treatment protocol provides a description of the processes and procedures involved in the acupuncture treatment. [file 1471-2474-7-99-S1.doc]

# Treatment protocol

The treatment protocol was developed by three of the study acupuncturists and Hugh MacPherson:

###### The following list of processes and procedures provide a structure for the key aspects of treatment. There is considerable flexibility, in order that the treatment can match patient variability. The protocol is not too broad, such that “anything goes” as this would mean that it would be more difficult to attribute any impact of treatment to acupuncture. The protocol is also not too narrow, as we want the treatments to broadly reflect how acupuncturists normally work. What follows are a series of processes and procedures:

1. Take the case history, including current orthodox medical diagnosis and treatment, and undertake traditional acupuncture diagnostic assessments, by asking questions, palpating, observing, smelling and hearing.

2. Identify your patient’s patterns of disharmony from within your normal repertoire of traditional acupuncture diagnostic frameworks.

3. Implement your treatment strategy based on your traditional acupuncture diagnosis, to be applied flexibly at each treatment on an individual patient basis.

4. In devising the treatment strategy, draw on diagnostic and treatment approaches that are integral to one or more of the theoretical frameworks identified in Table 1.

5. Determine a group of acupuncture points for needling, to be adapted as necessary at subsequent sessions, drawing on relevant theoretical considerations.

6. Select acupuncture needles of an appropriate length and gauge, based on relevant theoretical considerations.

7. Administer acupuncture needles to an appropriate depth, obtaining de qi where required, based on relevant theoretical considerations.

8. Use appropriate needling techniques, based on relevant theoretical considerations.

9. Retain needles for an appropriate period of time, based on relevant theoretical considerations.

10. Integrate the relevant auxiliary interventions, see Table 2, into the treatment strategy, the rationale for each being based on the theoretical approaches identified above.

11. Integrate relevant prescribed self-help activities, see Table 2, into the treatment strategy, the rationale for each being based on theoretical considerations.

12. Integrate into the treatment strategy relevant life-style support, see Table 2, the rationale being based on Chinese medicine theory identified above.

13. Use relevant explanations to provide for your patient any necessary information about their condition, their traditional acupuncture diagnosis, the aims and methods of the acupuncture treatment, and their prognosis.

14. Devise with your patient a treatment plan that covers the number and frequency of treatments, based on theoretical considerations as well as on their needs and availability and within the maximum number of treatments funded.

15. Elicit and interpret patients’ reactions to acupuncture treatment, whether positive and negative, reassuring patients where relevant, and incorporating this information into the ongoing treatment process.

16. Discuss and agree reasonable expectations with your patient of treatment progress with acupuncture, in the context of other treatments that they might be receiving.

17. Make judgements about the potential limitations of acupuncture, and where appropriate, encourage patients to seek help from their GP or other health professional, especially where there are concerns about the seriousness of the symptoms.

**Table 1 Diagnostic and treatment theoretical frameworks**

| Channels (e.g. local, adjacent and distal points on affected channel) |
| --- |
| Eight Extraordinary Channels |
| Eight Principles (e.g. internal/external, hot/cold, etc) |
| Qi, Blood and Body Fluids (e.g. Qi and /or Blood Stagnation) |
| Pathogenic Factors (e.g. Damp, Heat, Cold) |
| Trigger point acupuncture |
| Zang-fu syndromes (e.g. Kidney deficiency) |

**Table 2 Auxiliary techniques, and life-style support**

| **Auxiliary techniques:** |
| --- |
| Acupressure |
| Ah Shi points |
| Other acupuncture-related massage techniques |
| **Life-style support related to:** |
| Rest |
| Relaxation |
| Exercise |
| Diet |
| Work |
